# Evaluation of Functional Correlation of Task-Specific Muscle Synergies with Motor Performance in Patients Poststroke

**DOI:** 10.3389/fneur.2017.00337

**Published:** 2017-07-19

**Authors:** Si Li, Cheng Zhuang, Chuanxin M. Niu, Yong Bao, Qing Xie, Ning Lan

**Affiliations:** ^1^Institute of Rehabilitation Engineering, Med-X Research Institute, Shanghai Jiao Tong University, Shanghai, China; ^2^Department of Rehabilitation, Ruijin Hospital of School of Medicine, Shanghai Jiao Tong University, Shanghai, China; ^3^Division of Biokinesiology and Physical Therapy, University of Southern California, Los Angeles, CA, United States

**Keywords:** muscle synergy, stroke, physiological index, reaching movement, motor performance, kinematics

## Abstract

The central nervous system produces movements by activating specifically programmed muscle synergies that are also altered with injuries in the brain, such as stroke. In this study, we hypothesize that there exists a positive correlation between task-specific muscle synergy and motor functions at joint and task levels in patients following stroke. The purpose here is to define and evaluate neurophysiological metrics based on task-specific muscle synergy for assessing motor functions in patients. A patient group of 10 subjects suffering from stroke and a control group of nine age-matched healthy subjects were recruited to participate in this study. Electromyography (EMG) signals and movement kinematics were recorded in patients and control subjects while performing arm reaching tasks. Muscle synergies of individual patients were extracted off-line from EMG records of each patient, and a baseline pattern of muscle synergy was obtained from the pooled EMG data of all nine control subjects. Peak velocities and movement durations of each reaching movement were computed from measured kinematics. Similarity indices of matching components to those of the baseline synergy were defined by synergy vectors and time profiles, respectively, as well as by a combined similarity of vector and time profile. Results showed that pathological synergies of patients were altered from the characteristics of baseline synergy with missing components, or varied vector patterns and time profiles. The kinematic performance measured by peak velocities and movement durations was significantly poorer for the patient group than the control group. In patients, all three similarity indices were found to correlate significantly to the kinematics of movements for the reaching tasks. The correlation to the Fugl-Meyer score of arm was the highest with the vector index, the lowest with the time profile index, and in between with the combined index. These findings illustrate that the analysis of task-specific muscle synergy can provide valuable insights into motor deficits for patients following stroke, and the task-specific similarity indices are useful neurophysiological metrics to predict the function of neuromuscular control at the joint and task levels for patients.

## Introduction

Stroke is the top three causes of death in aging population (1), and the followed disability has obliged a compelling medical and social need for rehabilitation (2). Among the impairments, motor dysfunction causes the most widely afflicted medical condition in patients suffering from stroke (3), especially that of the upper extremity due to its high usage in daily activities in life and non-stereotypical motor patterns (4). Even though substantial research efforts have been devoted to improving recovery (2), motor rehabilitation in the upper extremity is still a challenging issue because of limited understanding of the neurophysiological underpinning of recovery and lack of effective interventions (2, 5, 6).

One of the main issues in the rehabilitation of motor function is to assess the residual motor ability of patients quantitatively, so as to determine the amount of intervention necessary and to give precise guide in rehabilitation training. In clinical practice, measurements of kinematics and graded scores have often been used to estimate the overall ability of patients to accomplish daily tasks (4) and are adopted as outcome measures in clinical trials and research (7). The clinical scores commonly used in the evaluation of upper limb functions include Fugl-Meyer (FM) Score, Wolf Motor Function Test, and Motor Assessment Scale (8), which are based on performance outcomes of a set of required motor tasks. These scores lack the detailed information with regard to the ways that muscles and joints are controlled during a motor task (4, 9, 10). Since the motor task may be accomplished by a patient using the normal way (restitution), or using alternative strategies (compensation) (4), it is desirable that the assessment of motor ability can provide additional information that allows clinicians to determine the integrity of neuromuscular control in patients. This is particularly important in clinical intervention using multi-muscle functional electrical stimulation (FES) (11, 12).

To understand the control of the complex, redundant neuromuscular system (13), muscle synergy has been proposed as an optimized strategy by the central nervous system (CNS) (14–17), in which a group of muscles is activated in a specific spatiotemporal pattern (14, 15, 18–28). Muscle synergy allows the description of motor behaviors with a relatively limited set of muscle activation patterns (28). In this study, we adopted the “synchronous synergies” (9, 27, 29, 30), in which motor tasks are controlled by linear combinations of a few stereotyped sets of muscles (synergy vectors) that are activated simultaneously by corresponding temporal sequences (time profiles). Multiple computational approaches using factorization algorithms have demonstrated a robust synergy (31). Studies of upper limb and cyclic movements in healthy human subjects also revealed that muscle synergies are consistent across subjects (32–35).

Muscle synergy changes as new motor skills are acquired in infants with time (36), or with injuries in the CNS (37, 38), such as stroke (33), or in the peripheral neuromuscular system (29). Synergy analysis of a group of motor tasks in upper limb of stroke patients has indicated that nervous injuries might cause direct changes in the spatial connection or temporal activation of synergies (33, 39) and the followed adaptation may lead to merging or fractionation of synergy components in patients (39). A group of isometric force tasks in upper limb showed that muscle synergy differed in patients suffering from mild-to-severe stroke (40). It is also shown that there existed a correlation between clinical scores and performance of individual components of the muscle synergy during cycling in the lower extremity (41). These early studies strongly suggested that synergy analysis may be a potentially promising method for assessing motor functions in patients following stroke. Yet, questions remain as to how well a task-specific muscle synergy, such as reaching by the upper limb (6), could be a good metric for assessing neuromuscular control, task performance, and clinical outcome in hemiparetic patients. This is particularly relevant since task-oriented training (TOT) revealed a better recovery of motor function than non-task specific training (2, 6). Hemiparetic patients often had problems in reaching (42) due to abnormally high spasticity of muscles in the shoulder and elbow joints (42–44), especially in elbow extension (45). The structure of muscle synergy for a specific task may contain useful information on the residual ability, or deficits, of neuromuscular control in patients poststroke.

In this study, we hypothesized that there exists a positive correlation between task-specific muscle synergy and motor functions at joint and task levels in patients following stroke. The objective here was to establish a functional correlation between task-specific muscle synergy and performance at neuromuscular, joint, and task levels. To understand the relationship between normal and pathological synergy patterns, we developed a procedure to analyze synergies of forward and lateral reaching movements in age-matched control subjects and patients with hemiparesis. Both tasks required elbow extension and were highly used in daily activities of life. New similarity indices of synergy vectors, time profiles, and their combination were defined to represent quantitatively the relationship of pathological synergies of patients to the baseline synergy from control group. Analysis was carried out to correlate task-specific similarity indices with kinematics of joint movements, as well as the clinical FM score of patients. The task-specific muscle synergy is relatively simple to obtain clinically, and these quantitative metrics can be used in conjunction with clinical scores for assessing motor abilities and deficits in patients. More importantly, the pathological and baseline patterns of muscle synergies are useful in designing patient specific, assistive FES strategies for stroke rehabilitation (11, 12). Preliminary results of this study were also reported elsewhere in a conference proceeding (46).

## Materials and Methods

### Subjects

Ten hemiparetic patients with poststroke (S04–S13, 60.9 ± 6.6 years, nine males, detailed description in Table 1) were recruited from Ruijin Hospital of School of Medicine (Shanghai, China) for this study; they all suffered from moderate-to-severe impairment from ischemic stroke with a Fugl-Meyer score of upper limb (FMul) <50. The clinical scores presented in Table 1 were measured before the experiment by designated physical therapists. Patients had one of the following conditions were excluded from our study: spasticity higher than 1+ (MMAS); metal implant; cognitive difficulties; any other diseases that cause neurological impairment; and passive attitude with the experiment. Nine age-matched healthy subjects from the institute’s campus (H01–H09, 57.8 ± 5.9 years, five males, one left-handed) were recruited randomly as control. This study was approved by the Institutional Review Boards of Ruijin Hospital and the Ethics Committee of Human and Animal Experiments of the Med-X Research Institute of Shanghai Jiao Tong University. All subjects signed the form of informed consent before the experiment.

**Table 1:** Description of stroke patients recruited for this study.

Patient ID	Most affected side	Location of lesion	Months poststroke	BS	MMAS	FMul	FMarm
S04	Right	Left thalamus, right temporal lobe	5	IV	1	27	16
S05	Left	Brain stem, bilateral basal ganglia	5	IV	1	28	23
S06	Left	No significant lesions	10	IV	0	18	17
S07	Left	Right lateral ventricle, right frontal lobe	5	III	1	18	16
S08	Left	Right corona radiata	2	III	1	22	16
S09	Left	Right basal ganglia	3	IV	0	31	24
S10	Left	Right basal ganglia, right lateral ventricle, right frontal lobe	2	III	1+	21	20
S11	Left	Right pontine	2	III	0	32	21
S12	Left	Right lateral ventricle	3	IV	0	20	16
S13	Right	Left temporal, parietal and occipital lobe	2	II	1+	20	13

*BS, Brunnstrom Scale (I, no movement; IV, appear activities out of cooperative movement); MMAS, Modified Ashworth score for the elbow (0, no increase in muscle tone; 4, marked increase in muscle tone, affected part is rigid); FMul, Fugl-Meyer score of upper limb (full score of 66 for motor function in upper limb); FMarm, Fugl-Meyer score of arm (upper arm and forearm, full score of 44)*.

### Experiments

**Figure 1:**
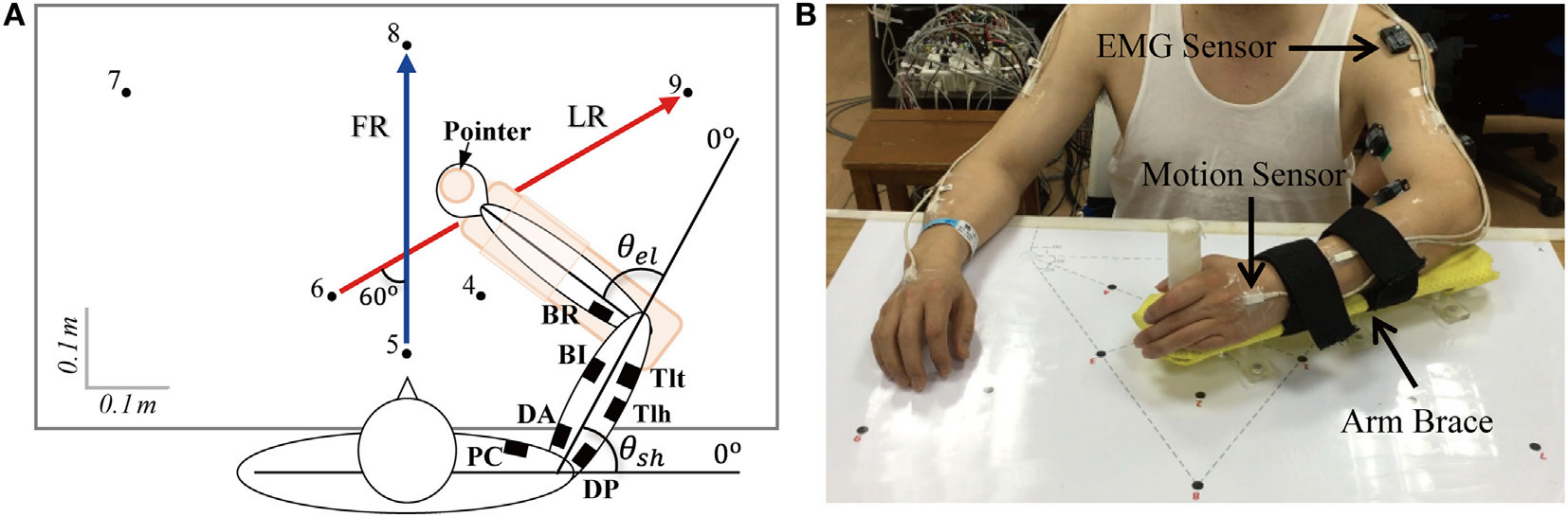
**(A)** Forward reaching (FR) and lateral reaching (LR) tasks and **(B)** experimental set up. Subjects moved the pointer from point 5 to point 8 when performing FR and from point 6 to point 9 for LR when the right arm was evaluated (point 4 to point 7 when the left arm was evaluated). Seven channels of electromyography (EMG) were recorded with wireless EMG sensors indicated by filled squares in **(A)**, the recorded muscles were pectoralis clavicular (PC), anterior deltoid (DA), posterior deltoid (DP), biceps (BI), triceps long head (Tlh), Brachioradialis (BR), and triceps lateral head (Tlt). *θ*_*_el_*_ and *θ*_*_sh_*_ are the angles of elbow and shoulder joints. Signals of position were collected by seven motion sensors (B), and they were put on bilateral upper arms, forearms, palms, and thorax (back of neck).

All subjects performed horizontal point-to-point reaching movements by their evaluated upper limb (affected hand of patient and dominant hand of control subject). As shown in Figure 1, subjects sat comfortably next to the table, with the trunk restrained with a corrective backrest to reduce its leaning forward and backward. The forearm was configured onto an arm brace on a smoothed motion plane. The hand was holding a vertical handle (pointer) with a diameter of 3 cm. The tasks included forward reaching (FR) and lateral reaching (LR). In FR, subjects moved the pointer from point 5 to 8 (36 cm), and in LR, the reaching was 48 cm from point 6 to 9 (right hand evaluated)/point 4 to 7 (left hand evaluated). The initial and terminal points were indicated by black dots on the horizontal motion plane with a diameter of 1 cm. Before the experiment, the subjects were trained to react to a verbal trigger and perform reaching as fast as possible without displacing their trunk. After practicing about five trials to make smooth reaching tasks, the recording started. During movements, no corrections were allowed and there was no feedback on their performance. Each task was repeated 10 times. A rest of 10 s in between trials and a break of 5 min between tasks were given for the subjects. During the experiment, positions of upper limb were captured at 120 Hz by seven magnetic motion sensors (Figure 1) with Motion Monitor II System (Innovative Sports Training, Inc., USA), and joint angles of shoulder and elbow were calculated from sensor signals. Electromyographys (EMGs) of seven muscles, including pectoralis clavicular (PC), anterior deltoid (DA), posterior deltoid (DP), biceps (BI), triceps long head (Tlh), brachioradialis (BR), and triceps lateral head (Tlt), were recorded at 1,925.9 Hz using the Trigno Wireless EMG System (Delsys Inc., USA), the isolated EMG sensors (37 mm × 26 mm × 15 mm) were placed center of each muscle belly under the guidance of therapists.

### Signal Processing

Data of the 10 patients and nine control subjects were pre-processed before synergy analysis. Kinematic data were low-passed filtered with a cutoff frequency of 10 Hz (10th order zero-lag Butterworth) and differentiated to obtain velocity. The time instant where the velocity of hand was 10% of its peak value was defined as the initiation and termination of movement (47). The reaction time was defined as the time period from the instant of verbal trigger to that of movement initiation. A bell-shaped velocity profile (48) was used to fit the hand velocity of subjects (with time length of twice the movement duration, centered on the peak) to a Gaussian distribution curve (Curve Fitting Tool, MATLAB 2012b; MathWorks Inc.), the coefficient of determination (R of bell-shape) was adopted as the goodness of fitting.

Before processing the EMGs, signals from the Motion Monitor system and the Delsys EMG system were synchronized with the trigger signal. The EMG was first notch filtered at 50 and 120 Hz and their higher harmonics (16th order zero-lag Butterworth) to eliminate the interferences of power line and magnetic transmitter of Motion Monitor II System. The EMGs were then demeaned and band-passed filtered between 20 and 400 Hz (48th order zero-lag Butterworth) to remove motion artifacts and high frequency noise. Filtered EMG signals were finally full-wave rectified and low-passed filtered at the cutoff frequency of 20 Hz (19th order zero-lag Butterworth) to obtain the EMG envelope. The signals were filtered with zero phase shift, and all processing were performed off-line by custom developed programs (MATLAB R2012b; MathWorks Inc.).

### Muscle Synergy Extraction

We computed task-specific synergy for FR and LR tasks independently. Non-negative matrix factorization (NNMF) algorithm (31, 49) was chosen here to extract synchronized synergy from recorded EMGs of seven muscles (28). The algorithm modeled muscle activities as linear combinations of a sufficient number of synergy vectors (muscle weight) with time profiles of muscle activation. The algorithm was applied to the data set of each subject (including patients and control subjects) to extract individual synergy, as well as a pooled data set from all control subjects to extract a baseline synergy.

For individual synergy extraction, EMG envelopes with a time length of twice movement duration, centered at the peak of hand velocity, were selected to construct EMG matrix. The synergy decomposition was given in the following equation:

(1) $$M_{\left(t\times 1,000\right)\times 7}=T_{\left(t\times 1,000\right)\times n}V_{n\times 7}+\text{residuals}$$

where *M* is the original EMG matrix with seven columns of EMG data, *t* is the number of trials with each trial resampled to 1,000 data points; *V* is the matrix of *n* synergy vectors, in which each row contains a combination of the seven muscles with different weights, each vector was normalized to have unit length during factorization, and *T* is the matrix of time profiles, in which each column contains the activation profiles corresponding to each row of vector in all trials. During the extraction, the number of synergy vector (*n*) was increased successively from one to seven, and for each iteration of *n*, the NNMF was repeated 25 times, the repetition with the lowest residuals of reconstruction was selected.

We defined the baseline synergy for each task as that obtained from pooled data of all nine control subjects. For each task, data of all trials from H01 to H09 were cascaded together to construct the pooled EMG matrix, and the baseline synergy was then computed from Eq. 1 using the pooled EMG matrix.

To evaluate the goodness of EMG reconstruction, the criterion of variance account for (VAF) (27, 29, 33, 39, 50) was adopted here in the following equation:

(2) $$\text{VAF}=1-(||M-D|{|}^{2}/||M-\text{mean }(M)|{|}^{2})$$

in which, *D* is the reconstructed EMG matrix; the operator “mean” constructs a matrix of the same size of *M* but with the elements of each row replaced by the mean value of corresponding row in *M*. The number of synergy vectors (*k*) that sufficiently recaptured the original EMGs was then defined as the minimum number (*n*) when VAF exceeded 80% (39) in more than half of the subjects in both groups. We checked the goodness of reconstruction of global and individual muscle’s EMG at *k* synergy components with another widely used criterion of variance account for (VAF′) (40, 41, 51) as well, which is sensitive to both shape and amplitude of the signals (50).

(3) $$\text{VAF}\prime =1-(||M-D|{|}^{2}/||M|{|}^{2})$$

### Similarities of Task-Specific Synergies

To quantify the overall similarity between the synergies of subjects and the baseline synergy for each task, we defined new similarity indices to evaluate the degree of matching (see Appendix in Supplementary Material for computational details). We first calculated a value of closeness of individual synergy vector and time profile in each subject with respect to those of baseline synergy as in previous studies (29, 41, 51). Referring to the maximal scalar product criterion (29), individual synergy vector of a subject was paired to one of the baseline vectors, which had the maximal value of scalar product with it. Closeness of vectors (*C_V_*) was defined as the scalar product of the paired vectors. The two corresponding time profiles were then identified as the same profile, with the closeness (*C_T_*) given by a shape symmetry index (52). In this study, we proposed three similarity indices, such as vector similarity (*S_V_*), time profile similarity (*S_T_*), and combined similarity (*S*_COM_), to evaluate the overall similarity of subject’s synergy to the baseline synergy. The similarity indices of *S_V_* and *S_T_* were calculated using the closeness of individual vectors (*C_V_*) and time profiles (*C_T_*), respectively, weighted by their contributions (eigenvalues) in the reconstruction of original EMG matrix (see Appendix in Supplementary Material for computational details). The combined similarity (*S*_COM_) was the average of *S_V_* and *S_T_*. The three similarity indices of task-specific synergy, such as *S_V_, S_T_*, and *S*_COM_, were subsequently used to analyze how good was the neuromuscular control in patients than in control subjects.

### Statistical and Correlation Analyses

Two-way ANOVA was performed to detect the difference in kinematics, closeness, and similarity indices for group (cross-task, namely the pool of FR and LR) and task (cross-group, namely the pool of patients and control subjects). Independent two-tailed two sample *t*-test was used to detect differences in kinematics, closeness, and similarity indices between tasks within each group and between groups within each task. Linear regression was carried out in each task to assess the correlation of similarity indices to functional performance, such as kinematics and clinical FM scores. Cross-task similarity indices ($S_{V}\prime$, $S_{T}\prime$, and $S_{\text{COM}}\prime$) were also obtained by averaging the similarity indices of the two tasks, such as FR and LR, which were also correlated to the clinical FM score. Such correlations may allow us to establish the functional relationship of task-specific similarity indices to performance outcomes assessed by kinematic measurements and clinical scores. The significance level in statistical and correlation analyses was set at *p* < 0.05.

## Results

### Kinematics and EMGs in Control and Stroke Subjects

**Figure 2:**
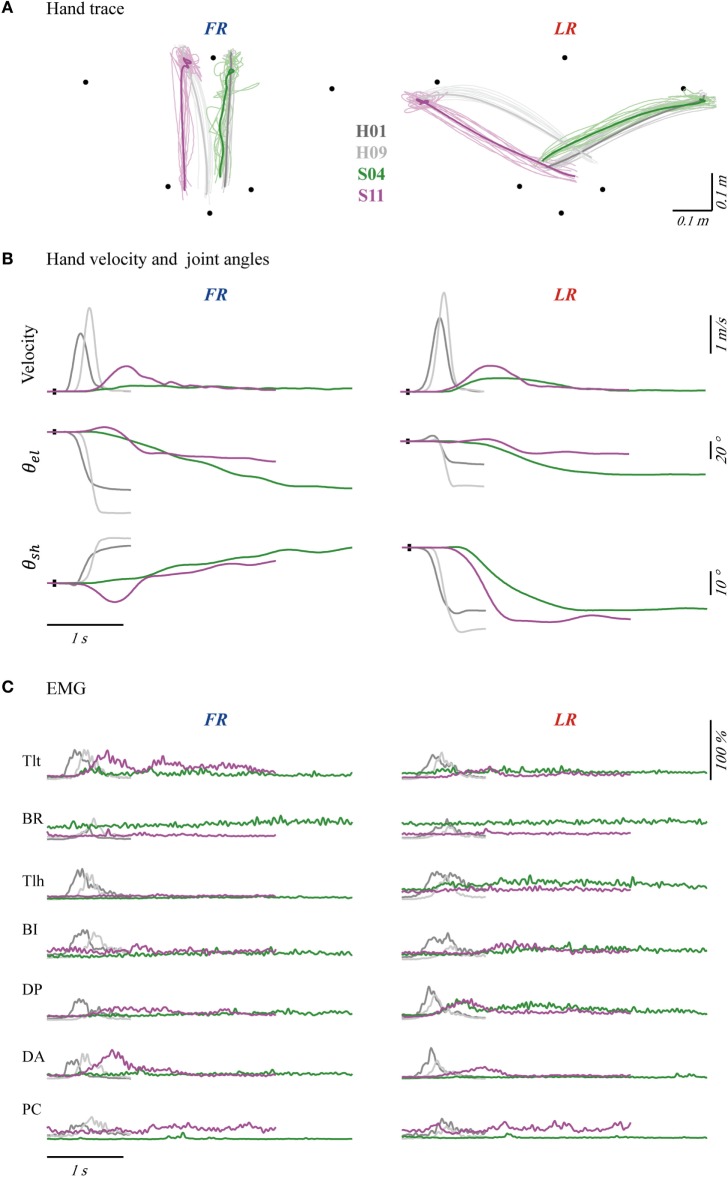
Hand traces **(A)**, hand velocities and joint angles **(B)**, and seven channels of averaged electromyography (EMG) **(C)** of four typical subjects, with forward reaching (FR) in the left column and lateral reaching (LR) in the right column. The bold profiles in **(A)** represent averaged trajectories. The black dots on profiles of velocity and angle indicate trigger tag. *θ*_*_el_*_ and *θ*_*_sh_*_ were angles of elbow and shoulder joint. Initial angles of shoulder and elbow were calibrated to mean of the four subjects’ angles. Each channel of EMG was normalized according to its maximal firing level among trials in each task. The EMGs were collected from pectoralis clavicular (PC), anterior deltoid (DA), posterior deltoid (DP), biceps (BI), triceps long head (Tlh), Brachioradialis (BR) and triceps lateral head (Tlt).

The kinematics and EMGs of two patients (S04 and S11) and two control subjects (H01 and H09) are presented in Figure 2. The synergy patterns of S04 and S11 were analyzed because they showed two extremes of performance in kinematics and clinical score (FMarm), as well as synergy. Comparing the two groups, control subjects showed a short reaction time, a smooth trajectory, and a classic bell-shaped velocity profile. However, the two patients showed a longer reaction time, a stagnated movement trajectory, and a multi-peak velocity profile, especially in FR (see for example in S04). For LR, both groups performed with higher speeds and smoother trajectories than for FR. The envelope of EMGs also exhibited intergroup differences. EMGs of H01 and H09 generally showed high bursting levels during movements and returned to steady state in a short period of time. In contrast, patients tended to activate their Tlt repeatedly in order to extend the elbow to reach to the final position. As shown in Figure 2C, both patients had weak firings of their Tlh, but a high background EMG in BR and PC, which impeded elbow and shoulder extensions. Patients also used a prolonged co-contraction of antagonistic muscles to stabilize joints after reaching the destination.

**Figure 3:**
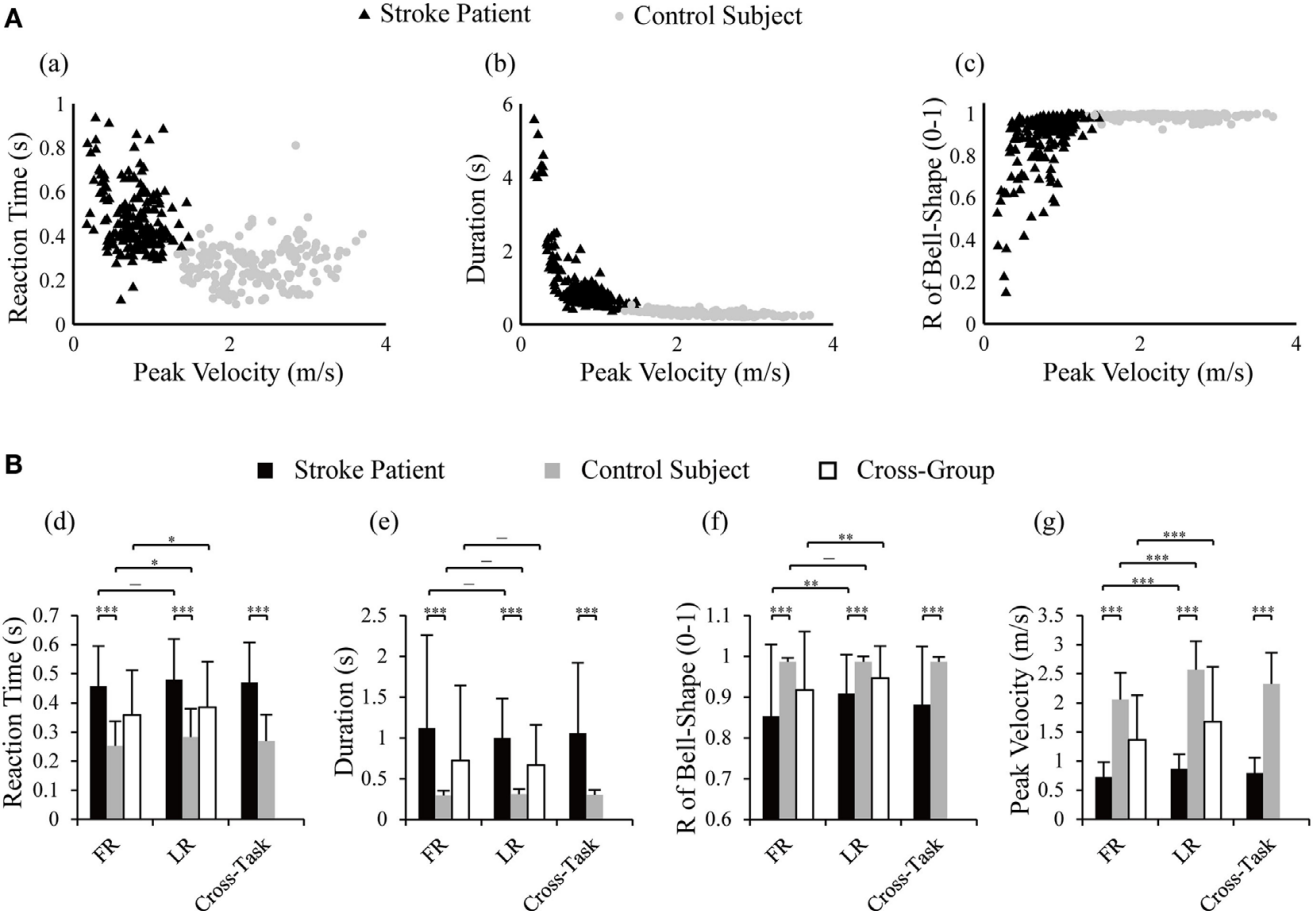
Distribution of reaction time, duration, and R of bell-shape with peak velocity in control subjects and stroke patients **(A)** and statistical comparison between the two groups and tasks **(B)**. Two-way ANOVA was performed with the factor of group (cross-task) and factor of task (cross-group); two-tailed two sample *t*-tests were used to detect differences in kinematics between tasks within one group and between group within one task; *p < 0.05, **p < 0.01, ***p < 0.001. R of bell-shape represents the coefficient of determination in fitting the velocity profile to Gaussian distribution curve.

Distribution of cross-task kinematic parameters in the two groups of subjects is plotted in Figure 3A. Separated distributions between patients and control subjects in reaction time, duration, R of bell-shape, and peak velocity could be visually recognized. Statistical analysis was performed to detect the differences of kinematics between tasks and groups. When comparing the two groups, significant difference was found in the four kinematics for each individual task (two-tailed, two sample *t*-tests) and the cross-task (two-way ANOVA). More specifically, patients possessed longer reaction time, longer duration of movement, lower R of bell-shape, and smaller velocity (*p* = 0.000 for the four parameters in FR, LR, and cross-task). The larger variability of duration and R of bell-shape (*p* = 0.000 for the two parameters in FR, LR, and cross-task) in patients reflected the varying degree of motor functional deficits. Two-way ANOVA also showed that kinematics except duration were significantly different between the two tasks with the cross-group (Figure 3B). LR displayed longer reaction time (*p* = 0.028), larger velocity (*p* = 0.000), and higher R of bell-shape (*p* = 0.008). This is in accordance with better kinematic profiles of LR than FR in Figures 2A,B.

### Muscle Synergies in Control and Stroke Subjects

In this study, we adopted the 80% VAF criterion (39) in the extraction of task-specific synergy in both control subjects and poststroke patients. For the baseline synergy from nine control subjects, the global VAF was 82.39% for FR with a three-component synergy and 89.95% for LR with a four-component synergy. As for individual subjects, global VAF of all subjects exceeded 80%, except for one case (S06) with a VAF = 74% in LR. When the number of components was increased to four for FR or five for LR, respectively, the improvement of VAF was less than 5%. Thus, we adopted the three-component synergy for FR and the four-component synergy for LR. We calculated the goodness of reconstruction with VAF′ as well (Eq. 3) (40, 41, 51). In both baseline and individual synergies, the global VAF′ for FR at three components and LR at four components were over 94%. In baseline synergy, VAF′ of individual muscle revealed the average of 88 (±13)% for FR at three components (VAF′ of six muscles exceeded 75%, except for muscle BR at 69%), and 90 (±12)% for LR at four components (VAF′ of six muscles exceeded 75%, except for muscle PC at 73%). For synergies of individual subject in the two groups, VAF′ of individual muscle was 93 (±2)% for FR at three components and 94 (±4)% for LR at four components.

**Figure 4:**
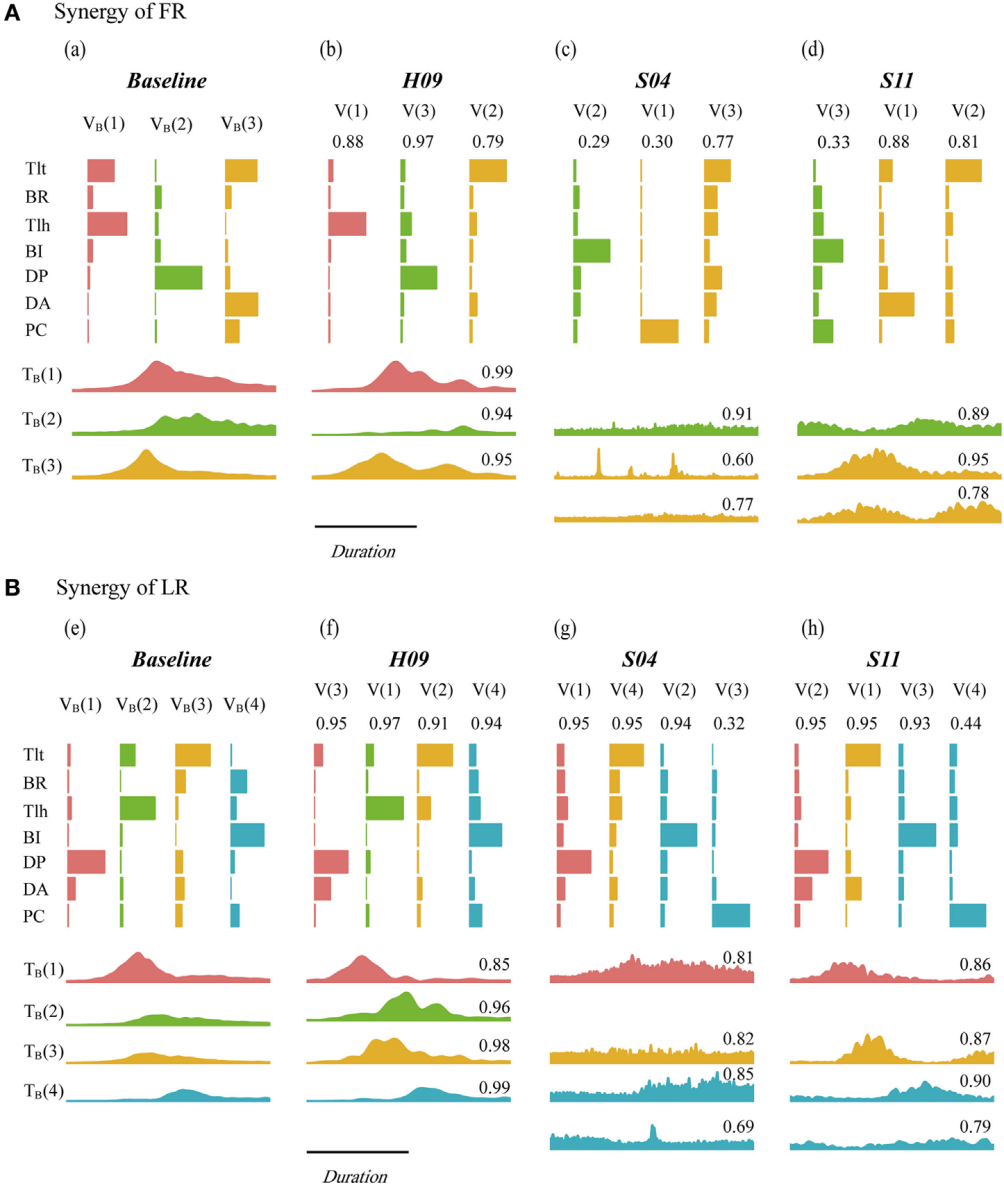
Synergy of baseline pattern, H09, S04, and S11 in forward reaching (FR) **(A)** and lateral reaching (LR) **(B)**. *V_B_* and *T_B_* are the vector component and time profile of the baseline synergy from the nine control subjects of the control group. *V* and *T* are the vector component and time profile from individual subject. Paired synergy vectors were plotted with same color for each task, the corresponding time profiles were presented successively under vector plots within each subject. The value on top of each vector and time profile indicates the value of closeness of individual vector and time profile.

Figure 4 depicts the synergies of FR (Figure 4A) and LR (Figure 4B) for the baseline pattern, a control subject (H09), and two patients (S04 and S11). Matching components and closeness of H09, S04, and S11 were indicated above the vectors and time profiles (also listed in Tables A1 and A2 in Supplementary Material). The matched vectors and time profiles within a task were indicated with the same color. The value of closeness ranged from 0.00 to 1.00, with 1.00 representing the highest degree of resemblance. It was clear that the synergy of H09 (Figure 4, b,f) possessed all components of those in the baseline synergy (Figure 4, a,e) with a high degree of resemblance in spatial and temporal patterns in both tasks. However, the synergies of patients of S04 (Figure 4, c,g) and S11 (Figure 4, d,h) deviated significantly from the baseline synergy. In FR task, the Tlt- and Tlh-dominant components of *V_B_*(1) in the baseline synergy were missing in the synergies of both patients. The DP-dominant component of *V_B_*(2) was partially preserved by two subjects, with a low closeness due to the dominating of BI. Only the third component *V_B_*(3) was kept relatively intact. In LR task, the components *V_B_*(1), *V_B_*(3), and *V_B_*(4) were well preserved in the synergies of both patients. Only the second component *V_B_*(2) was missing from the synergies of S04 and S11. The missing component in the two patients could be explained by their weak activations of Tlt and Tlh. Component of *V*(3) in S04 and *V*(4) in S11 showed poor closeness to *V_B_*(4), probably due to spastic firing of their PCs. In both tasks, the time profiles of both patients exhibited lower closeness to those of the baseline synergy than H09.

The matching pairs and closeness values of each vector (*C_V_*) and time profile (*C_T_*) in the two groups of subjects are listed in Tables A1 and A2 in Supplementary Material for FR and LR, respectively (see Appendix in Supplementary Material). As shown in Figure 4, the closeness values for FR were ranked generally in the order of the control subject H09 (high), patient S11 (low), and patient S04 (lowest). But for LR, the closeness values in the two patients (S04 and S11) was comparable to those of the control subject (H09), comparing to those of FR.

Inter-task comparison of baseline synergy showed that *V_B_*(1) and *V_B_*(2) in FR had the closeness of 0.97 to *V_B_*(2) and *V_B_*(1) in LR, respectively. *V_B_*(3) in FR and LR had a closeness of 0.84. The high closeness values declared that FR and LR possessed similar pattern of the first three components. This could explain the phenomenon that patients missing *V_B_*(1) of FR often missed *V_B_*(2) of LR simultaneously (Figure 4, Tables A1 and A2 in Supplementary Material). *V_B_*(4) was an extra decelerating component required in LR, and it had been well preserved by subjects in both group [closeness of 0.94 averaged from highest closeness to *V_B_*(4) in each subject]. The difference in the first three components between FR and LR lies in contribution of each component and their timing of activation. *V_B_*(1) and *V_B_*(2) acted as accelerating and decelerating units in FR, respectively, while in LR, *V_B_*(1) and *V_B_*(2) were synergistic in extension of the joints. *V_B_*(3) in FR was activated first in FR to flex the shoulder and extend elbow, and in LR, it helped with the extension of elbow after shoulder extension by *V_B_*(1) of LR. The mean closeness in patients to *V_B_*(1), *V_B_*(2), and *V_B_*(3) were 0.70, 0.64, and 0.56 in FR and 0.84, 0.89, and 0.94 in LR, respectively.

### Statistical Analysis of Closeness and Similarity

**Figure 5:**
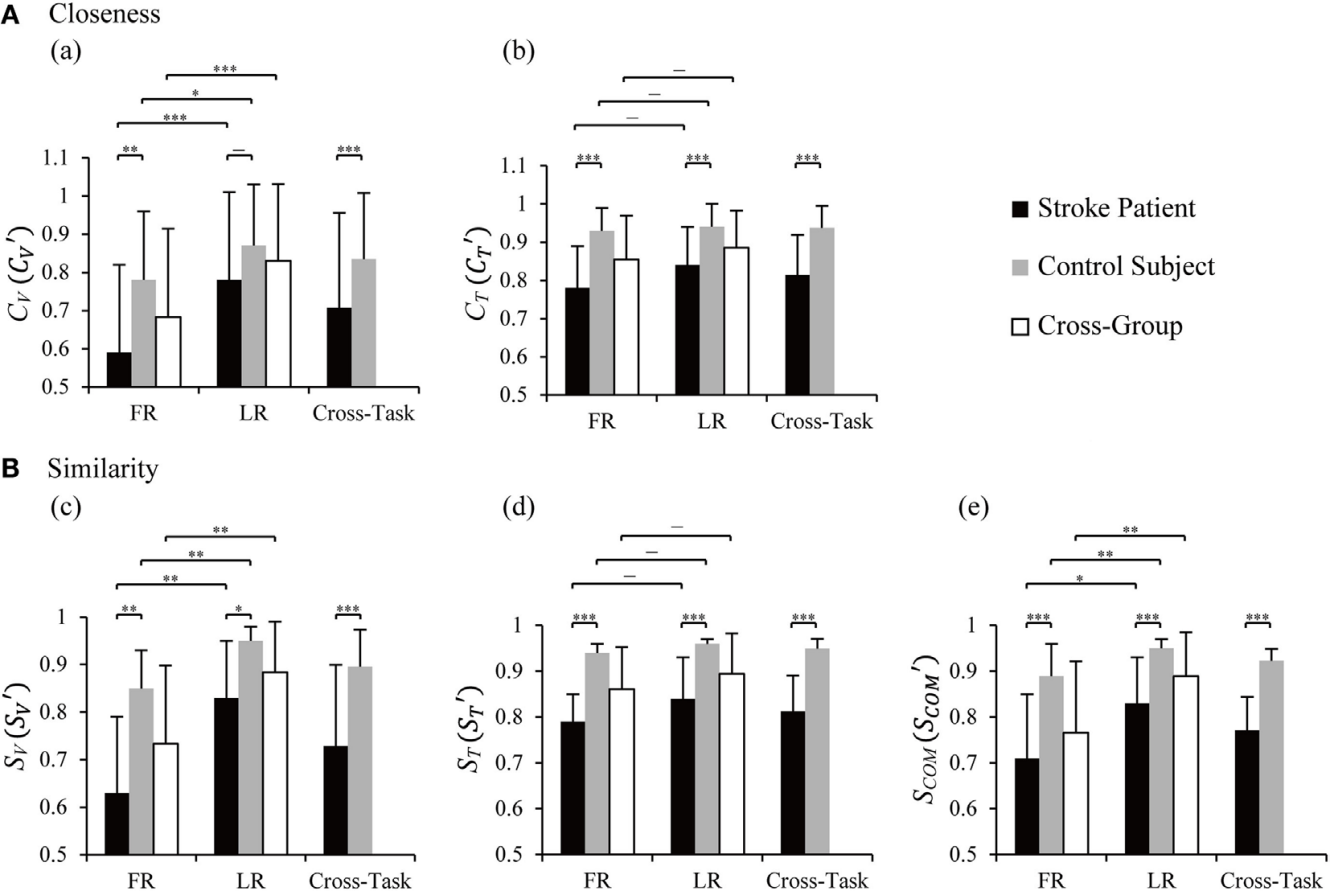
Statistical analysis of closeness **(A)** and similarity indices **(B)** between the two groups and tasks. *C_V_* and *C_T_* are closeness of individual vector and time profile, $C_{V}\prime$ and $C_{T}\prime$ are closeness in cross-task, and the values were averaged from *C_V_* and *C_T_* of the two tasks, respectively. *S_V_, S_T_*, and *S*_COM_ are similarity indices of vectors, time profiles, and their combination. $S_{V}\prime$, $S_{T}\prime$, and $S_{COM}\prime$ were cross-task similarity indices averaged from those of forward reaching (FR) and lateral reaching (LR). Two-way ANOVA was performed with the factor of group (cross-task) and factor of task (cross-group); two-tailed two sample *t*-tests was used to detect differences between tasks within one group and between group within one task; *p < 0.05, **p < 0.01, ***p < 0.001. Significant difference in similarity between FR and LR was found in *S_V_* and *S*_COM_ in patients [*p*(*S_V_*) = 0.007 and *p*(*S*_COM_) = 0.018], control subjects [*p*(*S_V_*) = 0.004 and *p*(*S*_COM_) = 0.003], and cross-group [*p*(*S_V_*) = 0.002 and *p*(*S*_COM_) = 0.006]. Significantly lower similarities in patients were found in FR [*p*(*S_V_*) = 0.002, *p*(*S_T_*) = 0.000, and *p*(*S*_COM_) = 0.000], LR [*p*(*S_V_*) = 0.020, *p*(*S_T_*) = 0.000, and *p*(*S*_COM_) = 0.001], and cross-task [*p*(*S_V_*) = 0.000, *p*(*S_T_*) = 0.000, and *p*(*S*_COM_) = 0.000].

Results of statistical analysis on closeness in all vectors (*C_V_*) and time profiles (*C_T_*) between groups and tasks are plotted in Figure 5, a,b. LR showed higher averaged closeness of vectors than FR in patients (*p* = 0.001), control (*p* = 0.044), and cross-group (*p* = 0.000). No difference in *C_T_* was found between FR and LR in patients, control subjects, and cross-group (*p* > 0.05). Between groups, patients presented lower *C_V_* and *C_T_* than those of control subjects in FR, LR, and cross-task, except for *C_V_* in LR (Figure 5, a).

To quantify the overall resemblance of muscle synergy of patients to baseline synergy, we defined more comprehensive similarity indices, a vector index (*S_V_*), a time profile index (*S_T_*), and a combined index (*S*_COM_) (Eqs A7–A9 in Supplementary Material). Statistical results (Figure 5B) indicated significant higher similarity of *S_V_* and *S*_COM_ in LR than FR in patients, control subjects, and cross-group (*p* values in legends of Figure 5). Between groups, similarity indices of *S_V_, S_T_*, and *S*_COM_ showed significantly higher values for control group than those for the patients in FR, LR, and cross-task (*p* values in legends of Figure 5). This result illustrated that the similarity indices were capable of distinguishing the different abilities of neuromuscular modulation in control subjects from those in patients.

### Correlation of Similarity Indices with Kinematics and FM Score

**Figure 6:**
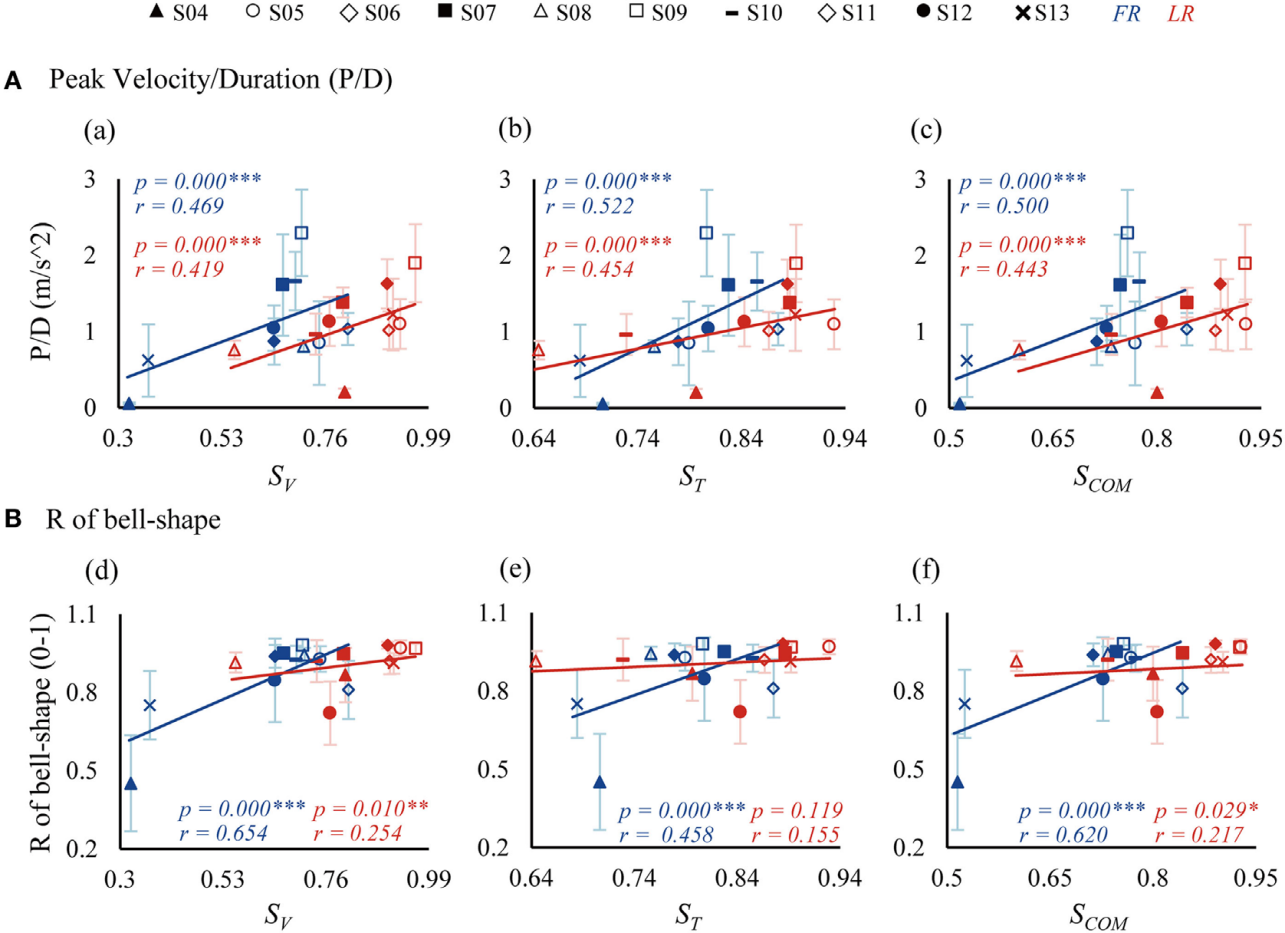
Correlations between similarity indices and kinematics [P/D in **(A)** and R of bell-shape in **(B)**]. P/D is the ratio of peak velocity and duration. R of bell-shape represents the coefficient of determination in fitting the velocity profile to Gaussian distribution curve. *S_V_, S_T_*, and *S*_COM_ are similarity indices of vectors, time profiles, and their combination.

**Figure 7:**
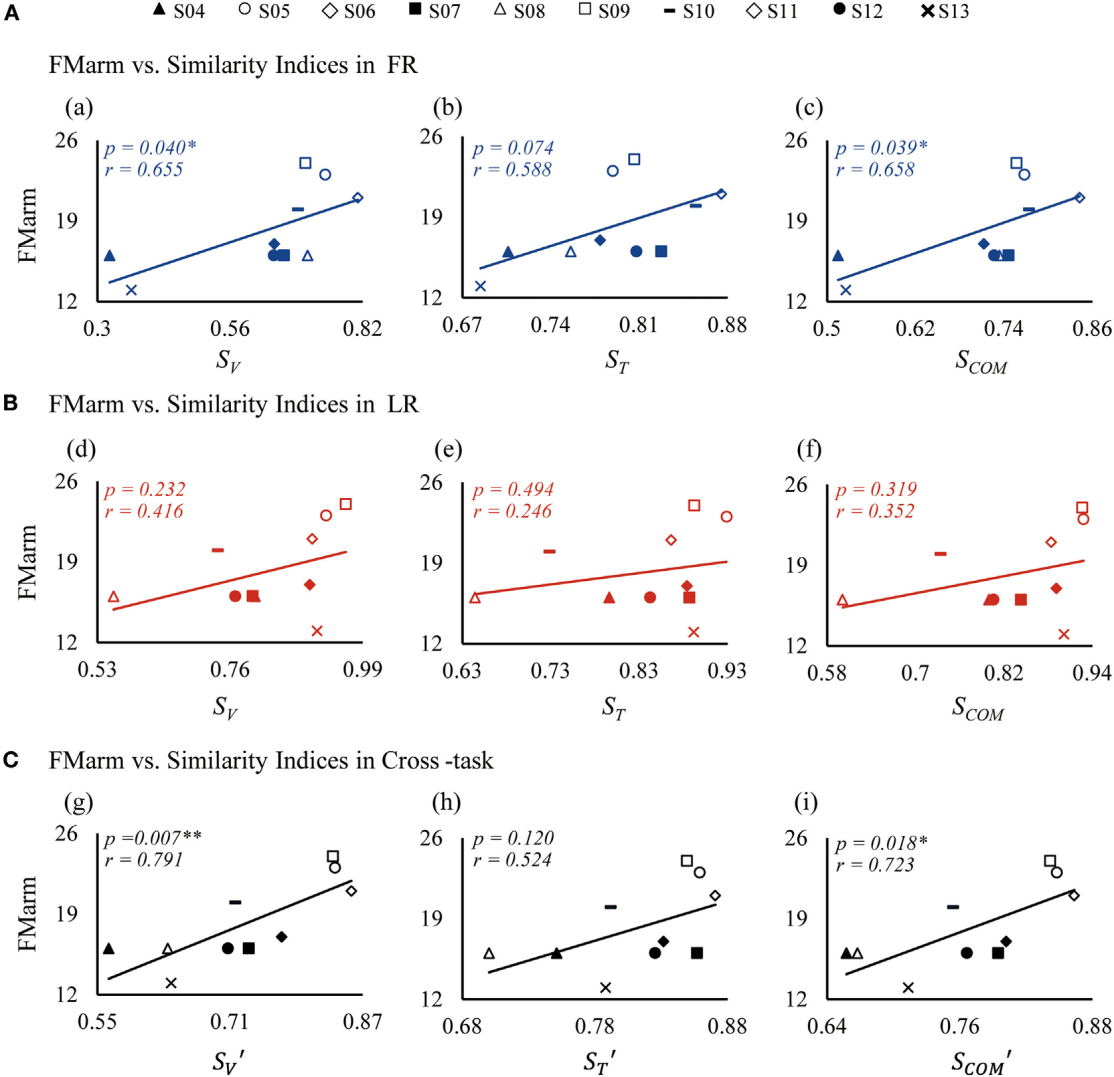
Correlations between similarity indices and FMarm score in forward reaching (FR) **(A)**, lateral reaching (LR) **(B)**, and the cross-task **(C)**. FMarm is Fugl-Meyer score of arm. *S_V_, S_T_*, and *S*_COM_ are similarity indices of vectors, time profiles, and their combination, respectively; while the indices of $S_{V}\prime$, $S_{T}\prime$, and $S_{COM}\prime$ are cross-task similarity indices averaged from those of FR and LR.

The similarity indices were correlated to kinematics of movements and clinical FM scores of patients, as presented in Figures 6 and 7, respectively. In general, significant correlations were found for the three similarity indices with respect to kinematic performance (Figure 6, significances were indicated in separated regressions), except for an insignificant correlation between *S_T_* and R of bell-shape in LR (Figure 6, e). Patients with a higher value of similarity indices tended to produce a better performance with a higher ratio of peak velocity and duration (P/D) (Figure 6A), and a better bell-shape profile (Figure 6B). Thus, the three similarity indices represent well the abilities of patients to control FR and LR tasks.

A relationship between patient FM scores and similarity indices is also clearly demonstrated in Figure 7. Since the recorded muscles were concerned with functions of the arm, the Fugl-Meyer score of arm (FMarm) was picked out from the Fugl-Meyer score of upper limb (FMul) as a factor for correlation analysis. For FR shown in Figure 7A, the FMarm score was found to have a significant positive correlation with *S_V_* (*p* = *0.040*) (Figure 7, a) and *S*_COM_ (*p* = *0.039*) (Figure 7, c). Only a weak correlation between *S_T_* and FMarm (*p* = *0.074*) was evident (Figure 7, b). For LR, however, the correlation of the FMarm was not significant for all three similarity indices (Figure 7B). This may be due to the fact that FMarm data points in LR were more scatted around the regression line (Figure 7B). Using the cross-task similarity indices (Figure 7C), a strong correlation of FMarm was evident, especially for the vector similarity $S_{V}\prime$ (*p* = *0.007*) (Figure 7, g) and the $S_{\text{COM}}\prime$ (*p* = *0.018*) (Figure 7, i). Nevertheless, a trend was clearly displayed in that patients with a higher FMarm score generally demonstrated a higher value of similarity indices. We also checked that no significant correlation existed between all similarity indices and the Fugl-Meyer score of upper limb (FMul).

## Discussion

In this study, we developed a computational procedure to evaluate task-specific synergies of reaching movements in stroke patients and age-matched control subjects. We found that three and four components were required to account for forward and lateral reaching movements, respectively. New quantitative indices of similarity of synergy in patients with respect to the baseline synergy were developed and employed to establish positive correlations to kinematic performance and clinical scores, such as FMarm. The results supported our hypothesis that there is a positive correlation between task-specific similarity indices and motor performance in joint and task levels in patients following stroke. This indicated that the new similarity indices based on task-specific synergy could be useful neurophysiological metrics in clinical evaluation to estimate motor dysfunction, or the ability of motor control in conjunction with clinical scores. The main contribution of this study is that we extended the analyses of muscle synergy (33, 40, 41) into quantitative metrics that may facilitate the clinical evaluation of patient’s motor functions with insights into neuromuscular control.

### Task-Specific Muscle Synergy

We focused on task-specific synergy in patients and demonstrated that the synergy analysis of a specific task could provide valuable insights into deficits in motor functions. TOT has been widely encouraged in stroke rehabilitation (2). Under certain requirements, patients with motor dysfunction are activated to search for better solutions to motor problems (6), and TOT has revealed better recovery of motor function than unspecific task training (2). We chose reaching tasks because discoordination of joints and abnormal co-activations of muscles in upper limb (43, 44) often resulted in difficulty in performing reaching movements in most stroke patients (42). In particular, elbow extension was found to be an important predictor for motor function in patients (45). Thus, instead of pooling a set of arm movements together (33, 35, 39, 40, 53), we chose to examine the forward and lateral reaching movements for task-specific synergy evaluation. This approach could also be applied to other motor tasks that are relevant to clinical task-oriented interventions (2).

We obtained the task-specific baseline synergy as a target of comparison from pooled data of nine control subjects performing FR and LR tasks. Studies have shown that muscle synergies were robust across healthy subjects (32–34). Thus, we adopted the synergy extracted from dominant arms of the control group as an efficient baseline of synergy (54, 55) and compared synergy of the affected arm in patients to the baseline synergy. In our study, synergy baseline extracted from healthy control group presented similar muscle activations with previous studies (28, 32). To evaluate the degree of alteration in individual synergy component in subjects, we adopted scalar product (33, 41) to compute closeness between synergy vectors; a component of synergy of an individual subject was then matched with that of the baseline synergy giving the maximal value of closeness between vectors (29). Closeness of time profiles of paired synergy components was evaluated by a shape symmetry index using cross-correlation (51, 52). Thus, changes of synergy pattern in patients with respect to the baseline synergy could be quantitatively reflected by the values of closeness (Tables A1 and A2 in Supplementary Material). Statistical results showed significant higher closeness in control subjects than those in patients (Figure 5A). This confirmed altered muscle synergy in patients after cortical injury (39, 40).

Patients following stroke often had missing components of the baseline synergy (Figure 4; Tables A1 and A2 in Supplementary Material). Yet, pathological synergies might still preserve some components of the baseline synergy. Merging in synergy vectors has been observed in patients following stroke (39, 56). This was evident in the task of FR in both patients. *V*(3) of S04 was the merging of the three components in baseline synergy (reconstruction closeness at 0.98), *V*(2) of S11 was the combination of *V_B_*(1) and *V_B_*(3) (reconstruction closeness at 0.90). This was in accordance with the finding by Cheung et al. (39), patients had more baseline components that were merged in residual components (S04, comparing with S11) usually showed poorer performance of kinematics and FMarm. This neural compensation may be due to plasticity taking place in the brain (57–59). In addition, identification found fractionation in LR in both patients (39), that *V*(3) of S04 and *V*(4) of S11 were differentiated from *V_B_*(4) of baseline synergy. These alterations of synergy vectors in muscle weights in patients shed light not only to the impairment in individual muscle control but also to the regroup of muscles by neural compensation in the brain, which are important indicators of recovery of motor functions after stroke (56).

### Similarity Indices and Correlation with Kinematic and Clinical Performance

We extended previous analyses of muscle synergy (33, 40, 41) into quantitative neurophysiological metrics by defining three new similarity indices with values ranging from 0 to 1 to evaluate the integrity of motor functions in patients. An index value of 1 might indicate a nearly normal motor functions, and an index value of 0 might imply a severe loss of motor functions. It is shown that the similarity scores of patients were significantly lower than those of control subjects in both tasks (Figure 5B). Significant correlations were found between similarity and kinematics, so as with the Fugl-Meyer score of arm (FMarm). In particular, the similarity indices of synergy vector *S_V_*, and time profile *S_T_*, showed good correlations with movement kinematics (Figure 6), indicating a strong causal relation of neural organization of muscle activation with motor performance (9). For FMarm score, its correlation was task sensitive. In FR, the similarity index of vector *S_V_* was well correlated to clinical score of FMarm (Figure 7A). This suggested that *S_V_* could be a good estimate of the residual ability of muscle coordination in the execution of motor tasks by patients (40, 41). Cross-task indices that combined the similarity indices of the two tasks, time profile ranked the worst amongst all three indices in the correlation with FMarm (Figure 7C). The weak correlation between *S_T_* and FMarm may result from the fact that the clinical score of FMarm is often assessed by the final outcome of task performance, while *S_T_* may be a good indicator of soundness of dynamic planning and execution of motor tasks in patients (9, 41).

It is interesting to note that in LR, there was not a significant correlation between FMarm score and all similarity indices (Figure 7B). In fact, baseline vectors of the two tasks were quite similar except for a forth component in LR, while patients showed higher closeness and similarity indices in LR than those of FR (Figure 5, a,c,e). This might arise from synergetic role of muscles in the extension of elbow and shoulder during LR, and in FR, it required the flexion of shoulder and extension of elbow at the same time. Previous study has indicated that the ability to cooperate elbow extension during reach was a significant predictor of motor performance (45). These implied that FR in which a larger range of elbow extension was required might be a task more challenging. In other words, similarity indices are task sensitive, and synergy performance of FR may better distinguish different levels of motor ability in patients with varying degrees of impairment.

### Methodological Consideration

In our study, the synergies were extracted from non-normalized EMG, for there is not a method of EMG normalization (60) that may best serve our purpose here. The method of maximal isometric voluntary contraction (MVC) is undermined by the question whether the measured MVC represents the real maximal activating level (60), and measurements in patients are probably affected by their varying degrees of motor deficits. This might bring a larger inter-subject variability (61). Also, EMG is often normalized to the peak or the mean value for a specific task (PEAK/MEAN) (62). Nevertheless, EMG variations between different tasks, and the same task collected at different recovery stages in the same patients could not be intuitively compared by the method of PEAK/MEAN (60). Considering our interest to analyze the difference of synergy between tasks in this study, we did not try to normalize the EMG. In future studies, a proper method of EMG normalization applicable for both healthy and stroke patients may be considered.

### Further Implications for Neurorehabilitation

Muscle synergy could also provide guidance to intervention strategy using multi-muscle FES. FES has been widely used in the rehabilitation training in patients poststroke (2), and benefits are obtained both in improvements of movement control and brain cortical perfusion (63, 64). Multichannel stimulation showed an appreciable enhancement of motor ability in affected arm of patients poststroke (11, 64). A spatiotemporal neuromodulation of electrical stimulation series derived from synergy patterns from healthy rats demonstrated a significant recovery of motor function of spinal cord injured rats (65), supporting the use of synergy-based electrical stimulation for rehabilitation of motor control (59, 66). Our earlier study also explored the feasibility of applying synergy-guided electrical stimulation to the rehabilitation of motor control in patients poststroke (67). A synergy-based FES strategy was adopted in a TOT training procedure for patients following stroke using previously developed multichannel FES system (11, 12). Personalized intervention could be designed for each patient (12). It is promising to apply synergy-based approach in the assessment of motor functions and in the intervention of motor rehabilitation for patients poststroke.

## Conclusion

In this study, a computational approach to evaluate task-specific synergies of reaching movements was established that may be applied to clinical evaluation of motor functions of patients following stroke. New quantitative indices of similarity of synergy of patients were evaluated to establish positive correlations to kinematic performance and clinical scores. Our results illustrated that muscle synergy patterns contain rich information in their spatial components and temporal profiles. Comparing pathological synergies of patients to the baseline synergy can reveal deficits in the underlying neuromuscular coordination and control in patients suffering from stroke. The similarity indices based on such comparisons were found to relate well the individual ability of patients in task control to their kinematic performance and clinical scores of assessments. The analysis of task-specific muscle synergies should offer both researchers and clinicians new insights into the impairments in the neural organization of motor control in patients following stroke. The similarity indices may be useful neurophysiological metrics to evaluate deficits in motor functions and outcome of rehabilitation in conjunction to clinical scores.

## Ethics Statement

This study was approved by the Institutional Review Boards of Ruijin Hospital and the Ethics Committee of Human and Animal Experiments of the Med-X Research Institute of Shanghai Jiao Tong University.

## Author Contributions

SL designed and performed the human experiments, analyzed the experimental data, and prepared the figures and tables and the manuscript. CZ contributed to human experiments and data analyse. CN contributed to giving intellectual suggestions on experimental design, recruiting patient, and performing experiments. YB contributed to clinical measurement and caring of patients during experiments. QX contributed to assigning clinicians and configurations of patients’ experiments and giving constructive suggestions to the study from clinical point of view. NL conceived the human experiments, proposed the analytical and displaying methods, and edited the final version of manuscript.

## Conflict of Interest Statement

The authors declare that the research was conducted in the absence of any commercial or financial relationships that could be construed as a potential conflict of interest.
