# Supplementary material for: Evaluation of Functional Correlation of Task-Specific Muscle Synergies with Motor Performance in Patients Poststroke
Source: Front Neurol. 2017 Jul 19;8:337. doi: 10.3389/fneur.2017.00337 (PMC5516096; doi:10.3389/fneur.2017.00337)
Supplement: Supplementary file 1 [file Data_Sheet_1.pdf]

Appendix

Closeness of Individual Synergy Vector and Time Profile

Once the number of sufficient components of synergy (k) in each task was determined according to the VAF criterion of Eq. 2, the individual synergy vector in each patient were compared to those of the baseline synergy pattern. We defined a closeness matric of the synergy vector, as shown in Eq. A1,

$$X_V(i,j) = V(j) \cdot V_B(i) / (|V(j)| \cdot |V_B(i)|) \quad (i,j = 1,2,\dots,k) \tag{A1}$$

in which, X_V ($k \times k$) is a closeness matrix of synergy vectors defined by scalar product of V and V_B (33,41), V and V_B are vectors from individual subject and baseline synergy respectively. Thus X_V represent the closeness of each synergy vector in individual subject to the synergy vectors of baseline synergy, elements in X_V ranged from 0.00 to 1.00, with 1.00 representing the highest degree of closeness between vectors.

Therefore, each column of X_V consisted of closeness between a vector from individual subject and the k vectors in baseline synergy, and the vector from individual subject was then paired to one from baseline synergy when they had the maximal closeness among the k values (maximal closeness principle) (29). The definitions were in Eq. A2-A3,

$$N(i) = \max(X_V(1,i), X_V(2,i), \dots, X_V(k,i)) \quad (i = 1,2,\dots,k) \tag{A2}$$

$$C_V(i) = X_V(N(i), i) \quad (i = 1,2,\dots,k) \tag{A3}$$

$$C_T(i) = |I(\tau)| / \sqrt{\sum_{m=1}^{1000} \overline{T(i)}^2(m) \cdot \sum_{m=1}^{1000} \overline{T_B(N(i))}^2(m)} \quad (i = 1,2,\dots,k) \tag{A4}$$

where operator "*max*" was meant to obtain the maximal value of closeness among the ith column of X_V. *N(i)* returns the serial number of component in baseline synergy, which has the highest value of closeness of individual vector to the ith vector in each subject. C_V was then defined as closeness of individual vector (Eq. A3). The corresponding time profile of paired vectors were then identified as the same time profile, their closeness was given by the shape symmetry index between $\overline{T}$ and $\overline{T_B}$ (52) (Eq. A4), $\overline{T}$ and $\overline{T_B}$ are averaged time profiles from individual subject and baseline synergy respectively. $I(\tau)$ is the circular cross-correlation function with a time-lag τ between the two profiles, and τ is set to zero in our evaluation. The values of closeness for synergy vectors and time profiles obtained in this study are enlisted in Table A1 & A2.

Similarity Indices for Task-Specific Performance of Synergy

We defined three similarity indices to give an overall evaluation of task-specific synergy performance. The indices were derived from weighted closeness of individual vector and time profile, and the

weighting factor was based on contribution of each synergy component to the reconstructed EMG matrix. The process was as follows in Eq. A5 & A6,

$$[e_1, \dots, e_k] = pca(\bar{T}) \quad \text{(A5)}$$

$$\lambda_i = e_i / \sum_{j=1}^{k} e_j \quad (i = 1,2, \dots, k) \quad \text{(A6)}$$

here $\bar{T}$ $(1000\times k)$ was the averaged time profile from all trials in individual subject, operator "*pca*" (Principal component analysis) represented obtaining eigenvalue (e) of each column (each component) in matrix of time profile ($\bar{T}$), λ was the contribution rate derived from e. Since NNMF has modeled synergy as unit length of vectors and ordered time profile with decreasing length, therefore, λ_i was the contribution of ith component to the reconstructed EMG matrix, the factorization of *pca* was realized in Matlab 2012b (MathWorks Inc.).

Similarities of vectors and time profiles were then obtained from weighted C_V and C_T respectively, as in Eq. A7-A9, S_V was similarity of synergy vectors, S_T was similarity of time profiles, S_{COM} was the index of combined similarity, which was averaged from S_V and S_T.

$$S_V = \sum_{i=1}^{k} \lambda_i \cdot C_{V(i)} \quad (i = 1,2, \dots, k) \quad \text{(A7)}$$

$$S_T = \sum_{i=1}^{k} \lambda_i \cdot C_{T(i)} \quad (i = 1,2, \dots, k) \quad \text{(A8)}$$

$$S_{COM} = (S_V + S_T)/2 \quad \text{(A9)}$$

By means of closeness and similarity indices defined above, the task-specific performance of synergy of each subject could be quantified. The concept of closeness of individual vectors and time profiles based on scalar product and the shape symmetry index respectively (Eq. A1 & A4) have been adopted in other studies of synergy comparison (29,40,41,51,52). A similar component between synergies have also be identified using the maximal closeness (29). However, in this study, we outlined a computational procedure to analyze the similarity of synergies for a specific task, and we proposed three new similarity indices to quantify the similarity of subject's synergy to the baseline synergy based on Eqs. A7-A9. This computational procedure and similarity indices could be useful methods for clinicians to evaluate motor functions of patients following stroke.

Table A1- Closeness of vector and time profile in individual subject, and the paired component in the baseline synergy in the task of forward reaching (FR).

**Subject ID**	**FR**						***Missing Component***
	$C_V(1)$ (N)	$C_V(2)$ (N)	$C_V(3)$ (N)	$C_T(1)$ (N)	$C_T(2)$ (N)	$C_T(3)$ (N)	
H01	0.83 (3)	0.85 (2)	0.59 (1)	0.97 (3)	0.88 (2)	0.80 (1)	-
H02	0.97 (2)	0.86 (1)	0.38 (2)	0.94 (2)	0.96 (1)	0.87 (2)	*3*
H03	0.99 (2)	0.82 (1)	0.93 (3)	0.91 (2)	0.91 (1)	0.98 (3)	-
H04	0.96 (2)	0.91 (1)	0.44 (2)	0.95 (2)	0.98 (1)	0.98 (2)	*3*
H05	0.88 (3)	0.72 (1)	0.59 (3)	0.91 (3)	0.97 (1)	0.93 (3)	*2*
H06	0.78 (3)	0.78 (2)	0.30 (1)	0.94 (3)	0.98 (2)	0.73 (1)	-
H07	0.78 (1)	0.70 (1)	0.94 (3)	0.99 (1)	0.94 (1)	0.94 (3)	*2*
H08	0.99 (2)	0.76 (1)	0.79 (3)	0.98 (2)	0.97 (1)	0.88 (3)	-
H09	0.88 (1)	0.79 (3)	0.97 (2)	0.99 (1)	0.95 (3)	0.94 (2)	-
S04	0.30 (3)	0.29 (2)	0.77 (3)	0.60 (3)	0.91 (2)	0.77 (3)	*1*
S05	0.90 (1)	0.26 (3)	0.87 (2)	0.87 (1)	0.55 (3)	0.80 (2)	-
S06	0.72 (3)	0.71 (3)	0.35 (3)	0.75 (3)	0.88 (3)	0.71 (3)	*1,2*
S07	0.75 (1)	0.36 (1)	0.91 (2)	0.87 (1)	0.76 (1)	0.83 (2)	*3*
S08	0.73 (3)	0.71 (3)	0.38 (3)	0.78 (3)	0.68 (3)	0.85 (3)	*1,2*
S09	0.72 (3)	0.77 (1)	0.38 (3)	0.79 (3)	0.90 (1)	0.66 (3)	*2*
S10	0.73 (3)	0.81 (2)	0.29 (3)	0.88 (3)	0.93 (2)	0.58 (3)	*1*
S11	0.88 (3)	0.81 (3)	0.33 (2)	0.95 (3)	0.78 (3)	0.89 (2)	*1*
S12	0.72 (3)	0.41 (3)	0.47 (3)	0.81 (3)	0.81 (3)	0.86 (3)	*1,2*
S13	0.30 (3)	0.27 (3)	0.83 (3)	0.75 (3)	0.60 (3)	0.74 (3)	*1,2*

C_V and C_T, closeness of individual synergy vector and time profile in each subject (Eq. A1-A4), ordered with the decreasing contribution to the reconstructed EMG (Eq. A5-A6); N, the serial number of the paired vector or time profile in baseline synergy. Statistical analysis of closeness between the two groups was presented in Figure 5(A).

Table A2- Closeness of vector and time profile in individual subject, and the paired component in the baseline synergy in the task of lateral reaching (LR).

**Subject ID**	**LR**								***Missing Component***
	$C_V(1)$ (N)	$C_V(2)$ (N)	$C_V(3)$ (N)	$C_V(4)$ (N)	$C_T(1)$ (N)	$C_T(2)$ (N)	$C_T(3)$ (N)	$C_T(4)$ (N)	
H01	0.99 (1)	0.94 (3)	0.93 (2)	0.78 (4)	0.98 (1)	0.98 (3)	0.96 (2)	0.98 (4)	-
H02	0.99 (1)	0.95 (2)	0.64 (4)	0.87 (3)	0.95 (1)	0.97 (2)	0.90 (4)	0.95 (3)	-
H03	0.99 (1)	0.96 (2)	0.96 (4)	0.91 (3)	0.97 (1)	0.96 (2)	0.97 (4)	0.93 (3)	-
H04	0.99 (1)	0.93 (4)	0.91 (2)	0.67 (3)	0.97 (1)	0.92 (4)	0.97 (2)	0.94 (3)	-
H05	0.99 (1)	0.89 (3)	0.51 (4)	0.46 (4)	0.95 (1)	0.95 (3)	0.96 (4)	0.71 (4)	*2*
H06	0.95 (4)	0.99 (1)	0.91 (3)	0.48 (4)	0.97 (4)	0.98 (1)	0.90 (3)	0.79 (4)	*2*
H07	0.95 (3)	0.99 (1)	1.00 (2)	0.60 (4)	0.96 (3)	0.96 (1)	0.97 (2)	0.91 (4)	-
H08	0.99 (1)	0.88 (3)	0.95 (4)	0.71 (1)	0.97 (1)	0.98 (3)	0.97 (4)	0.88 (1)	*2*
H09	0.97 (2)	0.91 (3)	0.95 (1)	0.94 (4)	0.96 (2)	0.98 (3)	0.85 (1)	0.99 (4)	-
S04	0.95 (1)	0.94 (4)	0.32 (4)	0.95 (3)	0.81 (1)	0.85 (4)	0.69 (4)	0.82 (3)	*2*
S05	0.98 (1)	0.92 (2)	0.67 (4)	0.50 (4)	0.96 (1)	0.84 (2)	0.83 (4)	0.89 (4)	*3*
S06	0.93 (4)	0.78 (2)	0.94 (3)	0.96 (1)	0.93 (4)	0.88 (2)	0.81 (3)	0.87 (1)	-
S07	0.98 (1)	0.58 (4)	0.93 (3)	0.52 (4)	0.90 (1)	0.88 (4)	0.87 (3)	0.81 (4)	*2*
S08	0.31 (1)	1.00 (1)	0.72 (4)	0.42 (4)	0.59 (1)	0.69 (1)	0.92 (4)	0.76 (4)	*2,3*
S09	0.94 (3)	1.00 (1)	0.96 (2)	0.89 (4)	0.95 (3)	0.83 (1)	0.86 (2)	0.94 (4)	-
S10	0.98 (1)	0.35 (1)	0.93 (4)	0.91 (2)	0.89 (1)	0.47 (1)	0.86 (4)	0.79 (2)	*3*
S11	0.95 (3)	0.95 (1)	0.93 (4)	0.44 (4)	0.87 (3)	0.86 (1)	0.90 (4)	0.79 (4)	*2*
S12	0.89 (4)	0.94 (3)	0.58 (1)	0.30 (4)	0.95 (4)	0.81 (3)	0.80 (1)	0.68 (4)	*2*
S13	0.88 (4)	0.99 (1)	0.74 (4)	0.86 (2)	0.86 (4)	0.95 (1)	0.88 (4)	0.86 (2)	*3*

C_V and C_T, closeness of individual synergy vector and time profile in each subject (Eq. A1-A4), ordered with the decreasing contribution to the reconstructed EMG (Eq. A5-A6); N, the serial number of the paired component in baseline synergy. Statistical analysis of closeness between the two groups and the two tasks was presented in Figure 5(A).
